# Psychopathology of organic brain disorders

**DOI:** 10.3389/fpsyt.2024.1443197

**Published:** 2024-12-16

**Authors:** Jorge Calderón, Pablo Toro

**Affiliations:** Department of Psychiatry, School of Medicine, Pontificia Universidad Católica de Chile, Santiago, Chile

**Keywords:** organic, brain, psychopathology, symptom, gestalt, neuropsychiatry

## Abstract

The concept of mental symptom is constructed considering not only the biological signal that determines it, but the multilayered causative factors related to intersubjective experience. However, specific brain damage might produce a set of symptoms expressed in a recognizable gestalt that helps to differentiate organic of psychogenic causation. The legacy of the theory of mental symptoms developed by German Berrios and the seminal work of Hughlings Jackson and Kurt Goldstein can contribute to this difficult task.

## Introduction

The field of psychiatry, intricately entwined with diverse disciplines from medicine to humanities, deals with the complexity of human mental health. In this multifaceted landscape, understanding mental disorders transcends simplistic categorizations, demanding an appreciation of subjective experiences and the interplay of biological, psychological, and sociocultural factors.

German Berrios, an epistemologist of psychiatry, and the Chair of the Cambridge School of Psychopathology challenges the notion of mental symptoms as solely biological entities, advocating for a theory of mental symptoms that include a multilayered construction of meaning (1). Central to this discourse is the notion of descriptive psychopathology, that provides a structured framework for understanding and documenting the complexities of mental disorders. However, the elusive nature of mental symptoms, shaped by personal narratives, cultural contexts, and clinician-patient interactions, complicates diagnostic precision. This complexity extends to the delineation of organic and functional etiologies, which, while serving as organizing principles, defy rigid boundaries in clinical practice. In fact, the heterogeneity of mental symptoms and more generally the difficulties in matching psychological language and brain activity have precluded the finding of reliable biological markers or pathophysiology of mental disorders (2).

Amidst these complexities, the legacy of pioneers such as Hughlings Jackson and Kurt Goldstein endures, offering invaluable insights into the holistic functioning of the brain and the profound impact of brain lesions on human cognition and behavior.

In the present work we will limit ourselves to structural brain damage that results in the manifestation of neuropsychiatric symptoms that seem to avoid (and only up to a certain degree) the impact of changes determined by personal semantic filter of symptom formation. A clinical approach to organic psychiatry and its psychopathology highlighted by the work of Jackson and Goldstein can inform clinicians about recognizable clinical features of structural brain damage, as one of many possible avenues for symptom formation explained by Berrios´s theory of mental symptoms.

## What is a mental symptom?

Psychiatry is a specialty of medicine that deals with mental disorders and is a heterogeneous discipline that requires the participation of different integrated approaches in the understanding of the patient’s subjective complaint and abnormal expressions of affect, cognition and behavior: neuroscience, psychology, sociology, among others. Descriptive psychopathology establishes a bridge between the different theoretical frameworks generating a way of recording the symptoms of a mental disorder, with a specific vocabulary, syntax and assumptions about the nature of behavior and some rules of application of these concepts (1).

From the perspective of Germán Berrios, the conceptual development of psychiatry is in a crisis that has repercussions in its clinical practice (1). These difficulties have their origin in the current predominance of the biological model that explains the formation of mental symptoms based on the analysis of the brain signal that determines them, as the main and/or only distinctive element. For Berrios, mental symptoms do not constitute natural entities but respond to hybrid descriptions in their formation and current configuration, and since the second half of the 19th century, fractions taken from the natural sciences (biomedical model) and contemporary human sciences have been integrated to understand and manage experiences and behaviors considered abnormal. The human sciences offer a privileged contribution if we consider that diagnosis in psychiatry generally involves more than the recognition of an ontologically stable object such as a tumor or other structural lesion of the body, most of the time undetected. Rather it is produced by the aggregation of different layers of significance to the assumed brain signal, which are constituted by aspects related to culture, personal and family narratives, the intensity and novelty of the signal among other aspects. This makes the mental symptoms often elusive, and always situated in each culture and period. Thus, it could be argued that psychiatry is a specialty whose language and conceptual development is not finished and may never reach a definitive development, if we consider that the abnormal will vary geographically and temporarily (3).

These difficulties in the clear demarcation of the mental symptom have forced clinicians to a complex procedure, which demands the analysis of the convergences, divergences and stability of the mental symptom through historical time, in its different structural aspects related to the word, the behavior and the concept that define it. The more stable in its construction, the more direct causal relationship this mental symptom will have with the biological signal that determines it. This will allow the development of more precise systems for capturing the symptom through an improved description (descriptive psychopathology), which in turn will be apprehended by other procedures such as functional neuroimaging, psychodiagnosis, evaluation scales, etc.

In clinical practice, the various mental symptoms have different importance in terms of their causal relationship with the biological signal that determines them. Some symptoms depend more closely on functional or structural alterations of the brain, others on aspects that are difficult to trace in the brain mapping, such as suffering derived from a frustrated expectation of success, for example. The mental symptom can elude the biomedical causality model because it is constituted in the interaction with the treating professional, where desires and/or intentions are expressed and crystallized in this exchange (4). For example, it could be the case that a patient is not willing (not consciously) to be relieved from a symptom that burdens him/her because of its relevance in the maintenance of a systemic-familial homeostasis. As a result, the symptom may be refractory to pharmacological therapy.

In the theory of mental symptom developed by Berrios and collaborators, the heterogeneity of the symptom depends on its construction process. The theory distinguishes primary construction processes with conscious (pathway a), or unconscious (pathway b) representations. In the case of pathway a, the biological signal undergoes a process of conceptualization by the patient that is determined by factors related to his or her previous experience, general knowledge, psychosocial determinants, etc. The signal initially produces an amorphous pre-cognitive material that is quickly endowed with meaning through personal and cultural codes, constituting a speech act that is expressed in communication with the clinician. In the case of pathway b, the signal evades conscious processing, and the symptom is expressed in such a way that it is recognized by the clinician in its form, without explicit reference by the patient to its presence. Its recognition will then depend on the clinician’s skills and knowledge. This theory admits secondary forms of construction, where the constructive process may not depend directly on the biological signal and arise from another symptom (phenocopy) (5).

In medicine the symptom refers to a subjective complaint of the patient in contrast to sign, which in turn is an observable attribute. In somatic disease this distinction is appropriate insofar as both share the same ontological nature as they are related to the same underlying process. In psychiatry, however, the separation between symptoms and sign is epistemologically impossible, since all signs have an expressive component that includes the subjective and that manifests itself in a meaningful whole.

The heterogeneity and complexity of the origin of the mental symptom means that old etiological demarcations such as the triad constituted by psychogenic, endogenous and exogenous causes have only a referential value. However, from the clinical point of view, the question about the ways of capturing the symptoms of pathway b, not conscious and therefore possibly subsidiary to a clearer biological signal, is relevant in that it depends to a greater degree on the clinician’s preparation for their detection and recognition. Even so, the recognition of the symptom will depend, even in the case that the biological signal avoids semantic configuration, not only on isolated abnormal behaviors, but on a structure of presentation that distinguishes it, and whose expression is framed in particular forms of the flow of consciousness. According to Jaspers, experience is never static or atomized and exists rather as a permanent and unitary becoming. The recognition of this holism distinguishes the structure or form of the symptom, essential for diagnosis in psychiatry (6). Therefore the notion of Gestalt understood as a salient unit representing the organization of the expressive phenomenon (symptom) helps us to grasp the organization of the phenomenon we configure as such, which is not only defined as an aggregate of different components but rather a whole where the whole is more than the sum of its (7). As an example, apathy and depression can be indistinguishable, as the share impaired volition and activity, although in the case of apathy, elements of sadness and anhedonia seem to be absent. In turn, in depression, gestalt is organized around an altered mood, and other complementary symptoms such as the presence of vegetative symptoms, anxiety and suicidal behavior (8).

If we consider that Berrios’ via b accounts for symptoms in psychiatry with a sharper biological signal, it is relevant to ask whether these types of symptoms have a clinical presentation that is distinguishable and differentiable from more semantically charged symptoms.

The debate about organic and functional (organogenesis versus psychogenesis) runs through the history of contemporary psychiatry. All attempts to define its boundaries via any form of dualism have failed. Functional disorders can lead to organic disorders. For instance, chronic depression that might have been triggered by psychological processes could lead to brain changes such as the reduction in hippocampal structures implicated in memory deficits associated to depression (9). Alternatively, when psychogenesis as opposed to organogenesis has been defined, as in the case of personality disorders, constitutional elements of personality defined through biological and/or heritable variables have been integrated into the causal model (10).

However, the distinction between organic and functional remains central to clinical practice, as an organizing principle of the various classificatory systems. Clinicians disagree on how to employ this distinction and report its use for strategic reasons and usually relying on a multiplicity of meanings assigned to these terms. It seems that one of the main functions in the use of this dichotomy has to do with ways of prioritizing interventions in favor of organic causes (11).

## Organic causation

The ways of approaching organic causation seem to vary substantially from one clinician to another, from causal attribution defined by the characteristics of the clinical presentation (apathy associated with cognitive deficits), deductive approaches based on the simultaneous presence of undifferentiated psychiatric symptoms, as emerging from a medical pathology (anxiety and insomnia in hyperthyroidism), or invoking a temporal relationship between the brain injury and the appearance of the psychiatric symptom (stroke and depression).

The distinctive features of the psychopathology of organic psychiatry represent a relevant source for the differential diagnostic process, as reflected in the opinion of Manfred Bleuler: “if the psychopathological study of a patient can demonstrate whether he is suffering from a brain disease or not, psychopathological findings cannot be ignored….I feel that it is one of the important tasks of psychiatry to teach the psychopathological differential diagnosis between mental derangements with and without brain pathology”. In this article, the author reviews studies of patients with brain tumors and their psychiatric manifestations, distinguishing an acute and a chronic presentation, the former characterized by compromised consciousness. Patients are drowsy and indifferent, with attention lapses and mood swings without obvious psychological precipitants. In the chronic presentation he distinguishes what he calls an amnestic syndrome, although he recognizes that this name does not account for the complexity of the presentation. Patients not only show memory difficulties, particularly recent memory, but suffer from a global deterioration of personality. There is a significant reduction in the content of thought, intellectual processes are markedly influenced by affective states and impulsivity, concepts become vague and unspecific, and the patient manages to grasp only one aspect of a given situation, neglecting other relevant aspects. There is a tendency to perseveration, confabulation, and emotional lability and fatigue. The patient responds only to what he/she understands easily, and shows a general lack of interest. Both presentations are unrelated to a specific tumor location (12).

At the end of the 19th century, academic discussion oscillated between localizationist and holistic paradigms in neurology. According to the localizationist hypothesis, the brain is made up of distinct modules that perform their function separately and can therefore suffer specific damage (13). This conception of the brain was criticized on the basis that it did not explain the recovery of the damaged function or the variability of the clinical presentation. In the holistic model the function was not circumscribed to a specific localization but was executed by a broad neuronal network. Prominent among the promoters of this holistic view of brain functioning in the late 19th and early 20th centuries were Hughglings Jackson and Kurt Goldstein.

Hughglings Jackson was a pioneer and founder of neurology (14). Influenced by the ideas of Herbert Spencer, he enunciated an evolutionary theory of brain function based on the existence of neural networks that are related in increasing complexity, allowing for better adjustment to adaptive demands. He proposes that the brain has evolved with new and more complex levels of re-representation of more basic sensory-motor representations, distinguishing three levels that integrate a complex sensory-motor unit such as the brain. The lowest level corresponds to the anterior horn of the spinal cord and motor nuclei, an intermediate level with the motor cortex and basal ganglia, and the highest level that re-represents the body, the prefrontal cortex.

Jackson argues that brain disease dissolves its hierarchical functions, generating two types of symptoms, those that are the consequence of the deficiency of the hierarchically superior function (negative symptoms), and those that reveal the expression of a lower hierarchical level manifested by the absence of inhibition of the center of higher evolutionary hierarchy (positive symptom).

Human consciousness expresses itself through representation, re-representation and complex recombination that allow the highest degree of integration in the processing of information that is essential for the development of reasoning, memory, emotions and behaviors.

Although these ideas were elaborated more than 100 years ago, they constitute an invaluable contemporary source for the development of cognitive neuroscience (15). As explained by Franz and colleagues, the model suggested by Jackson highlights the integration of higher-level representations that gather information from multiple inputs to generate an ideational representation that guide behavior, in a context mediated by different constraints. The re-representation of sensorimotor and perceptual data supposes the re- integration of contextual material and pre-existing patterns to elaborate a meaningful response to a complex situation.

They describe how inferences for Jackson´s model might allow the integration of contemporary brain scientists research in areas such as belief attribution, musical improvisation, and social cognition, among others.

For Goldstein, who was influenced by Jaksons’ model, developed his clinical and theoretical expertise with brain-damaged patients of World War I, diagnosis and therapy required preliminary consideration of the global changes in the patient’s personality and the impact of these changes on his or her environment (16).

While recognizing that the analytical method is the only legitimate scientific procedure for data capture, it is not useful for understanding the organism because isolation of function modifies the function being studied. The organism (brain) must be understood as a unit with phylogenetic characteristics independent of the “person”, which is more than a sum of parts.

Influenced by developments in Gestalt philosophy, he points out that the impact of brain injury must be analyzed in the context of the alteration of the normal process of oscillation between figure and background. For example, when raising the arm, an act that would constitute the figure, a disposition of the rest of the body is required to sustain the movement, which constitutes the background of this action. The brain lesion isolates the function and modifies the relation figure and background and forces the organism to a new adaptation or updating. This determines some changes in the organism, the reactions to the stimulus of the isolated part is exceptionally powerful, of an abnormal duration, linked more intensely to the external stimulus. The difficulty in the equalization of figure and background causes a greater lability and determines responses whose contents are more concrete. There is a modification of the function due to the brain lesion, but the function is not lost. The response threshold becomes changeable, which determines less predictability in the patient’s behavior, who becomes passive and dependent on external situations. Perseveration appears as a defense against impersistence, and catastrophic reaction as a non-conscious defense against the demands of actualization, which is expressed with greater behavioral instability, refusal to perform, or irritability. To avoid the catastrophic reaction the patient presents avoidance behaviors, isolation, exaggerated order, and lack of behavioral flexibility. For instance, a patient with dementia confronted with demands elicited by cognitive testing might initially feel at ease and then become agitated if unable to fulfill more complex tasks. This affective reaction goes normally unnoticed by the patient. It reveals the difficulties of the organism adapting to new demands that require abstract thinking. For Goldstein, the impact of the brain lesion determines the loss of the abstract attitude, which allows to voluntarily assume a certain mental attitude, to voluntarily pass from one solution to another, to simultaneously remember several aspects of a situation, to assimilate the essential of a set of aspects of a situation, to project, to adopt an attitude in relation to the possible, to delimit the self from the surrounding world (17).

The concrete attitude is pre-reflexive, realistic, in the sense that it anchors us immediately to the object, external situation, ideas, thoughts or emotions.

In the context of this difficulty to oscillate normally between the concrete automatisms and the abstract attitude (figure and background), the patient with a brain lesion loses certainty, speed, spontaneity and initiative. Affective changes often precede the more obvious cognitive changes. The patient appears superficial in his emotional reactions, indolent, irritable, querulous, and paranoid. The obsessional symptoms lack the usual distressing component of obsessions.

## Conclusion

As showed by McCaffrey et al. (18), the concept of functional localization has evolved, with contemporary challenges such as neural reuse, which had been foreshadowed by Goldstein (19), neural degeneracy, and contextual dependence of neural functions. The paper highlights the importance of reevaluating traditional approaches to functional localization in cognitive neuroscience considering contemporary challenges and the need for a more subtle understanding of structure-function relationships in the brain. Similarly, an anti-localizationism view is taken by Noble et al. (20), promoting the inclusion of complex systems and computational frameworks and multi-level explanations to capture the complexity of human brain. These attempts remind us of the relevance of holistic views of neural function described by Jackson and Goldstein based upon detailed clinical observations.

The model of symptom capture in psychiatry proposed by Berrios et al. considers the heterogeneity of the symptom in its expression and formation. A large part of the symptoms will become a speech act that crystallizes the pragmatic aspect of the relationship with the treating person, after going through a process of semantic and symbolic charge determined by the biological, psychological and social characteristics of the patient, and the novelty of the putative biological signal.

Other symptoms, however, will express themselves without the impact of this semantic filter, in a non-conscious way, and with a recognizable gestalt. Many of these symptoms are referred to changes in affective state and precede changes in cognition. The acknowledgement of these changes that affect the whole personality might be important for a premature differential diagnosis along with the presence of neurological soft signs (21).

## Data Availability

The original contributions presented in the study are included in the article/supplementary material. Further inquiries can be directed to the corresponding author.
